# Epidemiological Correlates of Substance Abuse Among In-Facility Clients of De-addiction and Rehabilitation Centres of District Dehradun: A Cross-Sectional Study

**DOI:** 10.7759/cureus.42826

**Published:** 2023-08-01

**Authors:** Nuzhat Zahin, Debabrata Roy, Richa Sinha, Sonam Maheshwari, Yashendra Sethi, Neil Patel

**Affiliations:** 1 Community Medicine, Government Doon Medical College, Dehradun, IND; 2 Medicine, Government Doon Medical College, Dehradun, IND; 3 Research, PearResearch, Dehradun, IND; 4 Medicine, Gujarat Medical Education and Research Society (GMERS) Medical College, Himmatnagar, Gandhinagar, IND

**Keywords:** addiction, substance abuse, socio-demographic variables, de-addiction centres, alcohol

## Abstract

Background

The increasing production, distribution, promotion, and availability of substances contemporaneous with the changing values of society have resulted in rising substance abuse as an emerging public health concern in India. A prevalence of 32-37% has been reported for substance abuse in various studies conducted in Uttarakhand but there is a dearth of data on socio-epidemiological factors affecting substance abuse.

Materials & methods

A facility-based observational cross-sectional study was conducted in selected de-addiction and rehabilitation centers of district Dehradun. Data were collected using multistage systematic random sampling from clients admitted to the facility.

Results

The mean age of in-facility participants was 28 ± 8 years and most of them started taking drugs after the age of 18 years. The most common substance of abuse was alcohol (61.7%) followed by tobacco smoking (15.6%). Both ‘peer pressure’ and ‘curiosity’ play a major role in predisposition to substance use. Further, we found that age (p=0.002), and level of education (p <0.001) were important determinants for substance abuse. At the same time, among other factors, the influence of occupation notably did not have a statistically significant association.

Conclusion

Sensitization and capacity building of both providers and the community is integral to effective strategizing for the prevention and control of substance abuse. Regional studies including the current study can be of help in framing drug policies and management guidelines including prioritizing the importance of the establishment of de-addiction and rehabilitation centers at the district level.

## Introduction

Substance abuse is persistent or sporadic drug use inconsistent with or unrelated to acceptable medical practice. It continues to be a major public health problem globally [1]. This issue has garnered greater attention recently, due to the changing trends in the usage, rather than the novelty or the magnitude of the problem [1,2]. A considerable percentage of individuals in India utilize psychoactive drugs (a chemical substance altering the function of the nervous system leading to changes in mood, perception, cognition, and behavior); the non-medical, non-prescription use of drugs is prevalent across all population groups. Alcohol is the psychoactive substance taken by Indians most frequently; 14.6% of the population between 10 to 75 years of age consume alcohol. Cannabis & opioids are the next most frequently consumed drugs in India after alcohol. Bhang (an edible preparation from cannabis leaves) use was found to be around 2%, while illicit cannabis products were about 1.2% [3]. Many regional surveys have been carried out in India to study the prevalence of substance use. The estimated prevalence of substance use in Uttarakhand was found to be 32-37% [3-6].

The published literature on the socio-epidemiological perspective of drug use in Uttarakhand is scarce. This facility-based observational study was designed to estimate the pattern, sociodemographic and behavioral attributes of drug use in district Dehradun, Uttarakhand covering the following aims: (1) to study socio-demographic & behavioral attributes of ‘in-facility’ clients of sampled de-addiction and rehabilitation centers of district Dehradun and (2) to study the pattern of substance abuse among study subjects.

## Materials and methods

Study design and study setting

An observational, cross-sectional study was conducted at select de-addiction and rehabilitation facilities of district Dehradun over a period of 12 months (August 2021 to July 2022).

Study population and subjects

The estimated population of district Dehradun abusing one or the other form of any substance was considered (5,93,843) [7] and 154 sampled in-facility clients undergoing de-addiction and rehabilitation at select facilities were selected for the study.

Sampling & sample size

Multistage systematic random sampling was employed. The sampling frame included all 56 randomly listed functional and documented de-addiction and rehabilitation centers of district Dehradun. Facilities sampled for the study comprised every fourth facility of the sampling frame by systematic random sampling (SRS) with a sampling interval (SI) of four amounting to 14 sampled facilities in total. Considering substance abuse prevalence in Uttarakhand to be an estimated 35 % [4-6], the sample size was calculated to be 151 with an absolute allowable error (L) of 8% and a rate of response of 90%. The number of clients chosen from each sampled de-addiction cum rehabilitation center of the district Dehradun was 151/14 = 10.78 ~ 11.

Inclusion and exclusion criteria

All the sampled in-facility clients older than or equal to 10 years of age who gave the consent were included, with the exception of clients who were under detoxification or those who denied consent for participation.

Statistical analysis

Data collection was done by using pre-structured and pre-tested proforma. All collected data were compiled, tabulated, and analyzed by using SPSS (BM Corp. Released 2013. IBM SPSS Statistics for Windows, Version 22.0. Armonk, NY: IBM Corp) & Microsoft Excel (2007). The frequency and percentage were calculated for all the variables. Yates’ chi-square test (Yates’ correction for continuity) was applied for association involving categorical variables to prevent overestimation of statistical significance for small data. The level of significance was assumed at p <0.05 and a confidence interval (CI) of 95%.

Ethical approval and informed consent

The study protocol was approved by the Institutional Ethical Committee (IEC) Government Doon Medical College, Dehradun, Uttarakhand. (Certificate no: GDMC/IEC/2022/REVISED/16). Data were collected in consensus with and approval of the clients after taking written informed consent. Consent for clients in the age group of 10 to 18 years was taken from either of the parents.

## Results

The study population (154 surveyed participants) were all males. The mean age of the participants was 28 (±8 SD) years, ranging from 17-57 years and mostly (58.4%) between 21-30 years of age. Most of the participants (90.9%) were Hindu, unmarried (70.1%), and about half of them (48.1%) were educated up to intermediate class. Table 1 illustrates the socio-demographic characteristics of the participants.

**Table 1:** Distribution of participants by socio-demographical characteristics (n=154)

	Frequency (n)	Percentage (%)
Age (in years)		
10 - 20	16	10.4
21 - 30	90	58.4
31 - 40	35	22.7
41- 50	8	5.2
51- 60	5	3.2
Gender		
Male	154	100.0
Female	0	0
Marital status		
Unmarried	108	70.1
Currently married	37	24.0
Others (separated, divorced, widowed)	9	5.8
Religion		
Hindu	140	90.9
Muslim	9	5.8
Others	5	3.2
Education		
Illiterate	3	1.9
Literate		
(a) Primary	7	4.5
(b) Upto middle school	15	9.7
(c) Intermediate	74	48.1
(d) Graduate	52	33.8
(d) Professionals	3	1.9
Occupation		
Student	34	22.1
Unemployed	35	22.7
Employed		
(a) Labour	32	20.8
(b) Business	32	20.8
(c) Service	21	13.6

Factors influencing substance use

Table 2 reveals that more than two-thirds of participants (68.2%) were first offered drugs by their friends or peers, and about one-fourth (26%) directly bought the drugs from dealers; interestingly, around 5.8% of participants were even offered drugs by their family members. About half (48.1%) of all participants stated that the ‘euphoric feeling’ induced by drugs was the ‘primary’ reason for initiating drug consumption, followed by peer pressure (26.6%). Only 7.8% of participants stated ‘family-related reasons,' including either negligence or family problems, as the main reason for starting drug intake. About 39.6% of participants used drugs in the company of a friend residing in the neighborhood, and one-third (33.8%) used drugs in solitude; the least common accomplice in drug use was a family member (0.6%). About three-fourths (72.7%) of participants ‘did not know/have any member(s) of their family who also consumed drugs, and the remaining 27.3% knew some other family member(s) using the substance/drug. Table 3 describes participants' distribution by substance use pattern (n=154).

**Table 2:** Distribution of participants by socio-behavioral attributes (n=154)

	Frequency (n)	Percentage (%)
Mode of introduction to substance		
Bought from dealer	40	26.0
Offered by friends	105	68.2
Offered by a family member	9	5.8
Other reasons	0	0
Apparent reason for starting substance		
To forget about family problems	12	7.8
Because friends are taking drugs	41	26.6
Drugs make us feel good	74	48.1
Negligence by family	6	3.9
Teenagers’ curiosity	20	13.0
To eliminate shyness	1	0.6
Accomplices in drug use		
Alone	52	33.8
With friends in neighbors	61	39.6
Friends at school	11	7.1
With friends at a party	29	18.8
With family members	1	0.6
Family history of drug consumption		
Yes	42	27.3
No	112	72.7

**Table 3:** Distribution of participants by pattern of substance use (n=154) LSD: Lysergic Acid Diethylamide

	Frequency (n)	Percentage (%)
Age (in years) of initiation of substance		
10-12	13	8.4
13-15	29	18.8
16-18	40	26.0
Above 18	72	46.8
Frequency of substance consumption		
Daily	104	67.5
Not daily but frequently	27	17.5
During weekends	13	8.4
On occasions	10	6.5
Substance used for the first time		
Cigarette/ Tobacco	24	15.6
Alcohol	95	61.7
Opium	10	6.5
Hashish	15	9.7
Ecstasy	1	0.6
Crystal	1	0.6
Morphine	1	0.6
Heroin	4	2.6
Cocaine	3	1.9
Others (crack, shireh (refined opium extract), LSD)	0	0

Socio-epidemiological correlates of substance abuse in Uttarakhand

The majority (46.8%) of the subjects indulged in substance abuse after 18 years of age. About two-thirds of the participants (67.5%) were found to be consuming drugs on a daily basis. The 'first’ substance abused by the participants was ‘alcohol’ (61.7%), followed by ‘tobacco smoking’ (15.6%). Less commonly used were ‘ecstasy’, ‘crystal’ & ‘morphine’ (0.6% each). As depicted inFigure 1, the most common substance abused was ‘alcohol’ (74%) followed by ‘tobacco’, mostly by ‘smoking’ (71.4%), then ‘opioids’ (46.8%) and ‘cannabis’ (40.3%). Three lesser common substances/drugs ever consumed by study participants were: ‘amphetamines’ (15.6%), ‘hallucinogens’ (14.9%), and ‘sedatives/sleeping pills' (11.0%).

**Figure 1:**
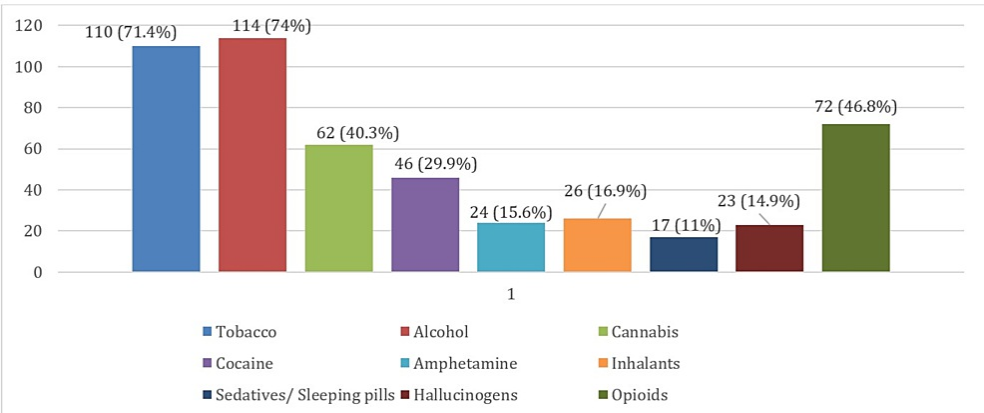
Distribution of participants by substance/drug ever used in life (n=154)

**Figure 2:**
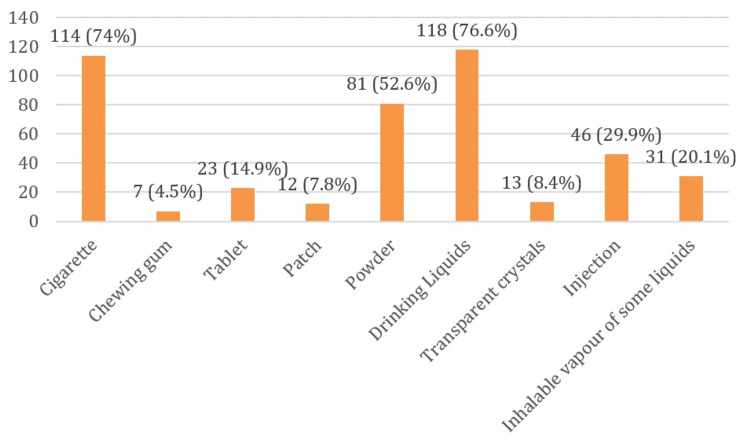
Distribution of participants by mode/form of drug consumption (n=154)

The single most common mode of consuming drugs was ‘ingesting’ liquid (76.6%) followed by ‘smoking’ (74%) and ‘sniffing’(52.6%); consumption of drugs in the form of ‘crystals’ (8.4%), ‘patches’ (7.8%) and ‘chewing gum’ (4.5%) were uncommon (Figure 2). From Table 4, it is evident that the difference in the proportion of clients according to age group between mono- and poly-substance abuse was statistically significant (p=0.002). Also, the level of education of the clients had a strong statistical association with the pattern of their substance use, i.e., differences in the proportion of mono- and poly-drug users clients for different levels of education was highly significant (p=0.00000144). Interestingly, the occupation of the clients was found not to be significantly associated with a pattern of substance use (p = 0.752).

**Table 4:** Association between variables of substance abuse.

Variables	Mono drug users	Poly drug users	Yates’ χ^2^ (Yates’ p-value)
Age Groups			
10-20	0 (0.0)	16 (12.0)	30.687 (0.002)
21-30	10 (47.6)	80 (60.2)	
31-40	7 (33.3)	28 (21.1)	
41-50	3 (14.3)	5 (3.8)	
51-60	1 (4.8)	4 (3.0)	
Education			
Illiterate	1 (4.8)	2 (1.5)	55.549 (0.00000144)
Primary	2 (9.5)	5 (3.8)	
Up to middle school	3 (14.3)	12 (9.0)	
Intermediate	7 (33.3)	67 (50.4)	
Graduate	7 (33.3)	45 (33.8)	
Professional	1 (4.8)	2 (1.5)	
Occupation			
Student	4 (19.0)	30 (22.6)	8.403 (0.752)
Unemployed	2 (9.5)	33 (24.8)	
Labour	6 (28.6)	26 (19.5)	
Business	6 (28.6)	26 (19.5)	
Service	3 (14.3)	18 (13.5)	

The results indicate the substance abuse pattern in Uttarakhand and contribute data on regional factors and socio-epidemiological correlates affecting substance abuse in the region.

## Discussion

Socio-epidemiological variables influencing substance abuse in a defined population vary with different study settings & parameters. The present study has distinct features distinguishing it from other analogous studies.

Numerous studies conducted in India and across the world have highlighted that drug addiction is a severe problem in the young adult population. Individuals in their second, third, and fourth decade of life are most often addicted to drugs. Similar to our findings (mean age of 28 years), Singh et al. [8] reported that the mean age of patients admitted to drug rehabilitation centers was 31.22 years, with the majority of patients being in the age group of 20-30 years (47.5%) followed by 30-40 years (35.8%). Similarly, other studies from other regions of India have reported similar mean age groups De Silva et al. [9] (34.04 years), Kadri et al. [10] (26-35 years), Gupta et al. [11] (31.22 years), and Majumder et al. [12] (21-30 years).

Interestingly, all of the sampled participants enrolled (154) in the present study were males (Table 1). There were no female in-facility clients at any of the 14 deaddiction centers surveyed, which is in line with findings of existing literature, including the National Family Health Survey-5 (NFHS-5), which suggests that all types of addiction are more common among males [13]. Analogous to our study, Randhawa et al. [14] and Arora et al. [15] also found all patients reporting to the government hospital settings in Punjab were male; Majumder et al. also reported that the majority of the substance users in their study settings were males (99.0%) [12].

Although all the participants enrolled and admitted to the deaddiction center were male, females could be addicted to drugs as well. Lack of awareness, greater social stigma, and negative cultural attitudes surrounding the usage of illicit substances by females, unlike in the case of males, may prevent females from reporting. The lack of female clients at the de-addiction centers can also be attributed to limited/fewer facilities meant for female clients, and poor health-seeking behavior due to gendered decision-making power and societal construct [12-16].

In the present study, 70.1% of participants were never-married, and only 24% of participants were married (Table 1). Corroborative to our findings, both Majumder et al. [12] and Rather et al. [16] reported that the majority of participants in their respective studies were never married. Contrary to this, Gupta et al. [11], Mohan C et al. [17], Mohan A et al. [18], Prajapati et al. [19], and Singh et al. [8] reported that drug abuse was more prevalent among married male participants. Randhawa et al. reported that an approximately equal number of patients were either married (53.21%) or single (46.79%) [14]. As most participants in our study were young adults, finding a large proportion of ‘never-married’ clients was not unexpected.

The study in context found that 90.0% of Hindus and 5.8% of Muslims were using one or the other form of the drug (Table 1). Besides the findings from NFHS-5 in India, i.e., 25% of Hindus and 6.3 % of Muslim populations use alcohol [13], very few studies are available with data on the religion-wise distribution of substance users. This could be explained by community hesitancy & sensitivity toward drug abuse vis-à-vis religion.

Most of the present study participants had school-level education (Table 1). Majumder et al. endorsed this study finding and reported that 46.7% of their clients had studied up to the school level [12]. Likewise, Rather et al. [16], and Gul et al. [20], had the majority of clients educated up to the school level. Contrary to this, a study found that most of the participants were graduates (49.3%) followed by high school education (24.2%) [21]. Clients’ profiles in terms of educational status may vary depending on their other socio-demographic attributes including study settings.

In the present study (Table 2), more than two-thirds of participants (68.2%) were first offered drugs by their peers, and about half of all (48.1%) enrolled participants reported ’curiosity’ for the drug as the ‘primary’ reason for initiating drug consumption. Saluja et al. [22], Wade et al. [23], So et al. [24], and Margoob et al. [25] had similar findings.

The next common reason (26.6%) was because their friends were consuming the drug. Only 7.8% of participants cited family-related reasons i.e., either negligence or family problems, as the main reason for starting drug intake. The present study also found that 39.6% of patients abused substances ‘with friends in the neighborhood’ and 18.8% of them with ‘friends at a party’ followed by 7.1% ‘with friends at school’, which suggests peer pressure influencing substance abuse. Likewise, Rather et al., reported that the commonest reasons for first use in their study were peer pressure and relief from a negative mood state [16]. Peer pressure has also been mentioned as a major initiating factor in previous studies by Venkatesan et al. [26], Gul et al.[20], and Margoob et al. [25]. It is widely recognized that there is a considerable link between peer pressure and the use of drugs and alcohol. Borsari et al. found that the peer environment is a factor in high-risk alcohol consumption through direct influences, modeling, and perceived norms [27]. Peer environments contribute to high-risk alcohol use. Kobus, in a comprehensive review, observed that the peer relationships of adolescents are a contributing factor to the smoking habits of adolescents [28]. Peer pressure was found to be the single most important factor in the commencement of substance use in a study that was conducted in Chandigarh [8] and Kashmir [29]. In this sense, our findings are consistent with what was found in past research. It may be inferred that both peer pressure and curiosity play a major role in predisposing to or determining drug use.

In the present study, a quarter of the patients gave a positive history of a family member using the drug. Gupta et al. [11], Kadri et al. [10], Singh et al. [8] had similar findings. Saluja et al. found that nearly half of the subjects had a positive family history of drug dependence [22], while Rather et al. [16] reported less than 10% had a positive family history of drug abuse. Evidence suggests that the family dynamic can either contribute to or mitigate youth social and behavioral difficulties.

The most common substance abused by the clients in the study (Figure 1) was alcohol (74%) followed by tobacco (mainly smoking - 71.4%), opioids (46.8%), and cannabis (40.3%), respectively. Prajapati et al. [19], Venkatesan et al. [26], and Kadri et al. [10] in their respective studies conducted in comparable but different settings reported that alcohol was the most common substance abused by patients admitted to drug de-addiction and rehabilitation centers and polysubstance dependence showing an increasing trend. Prajapati et al. [19], also reported that consumption of traditional drugs like opium and cannabis was remarkably low as compared to alcohol and tobacco. In contrast, Majumder et al. [12], Gul et al. [20], Rather et al. [16], and Saluja et al. [22] found that opioids were the most frequently used substance in their studies, clients using opioids ranging between 42.9% to 65.7%. A national-level survey undertaken by the Ministry of Social Justice and Empowerment in 2019 [30] mentioned that alcohol was the most common substance used in India; the two next most commonly used substances in India were cannabis & opioids; a sizeable number of people used other categories of substances like sedatives and inhalants. It is also noteworthy that alcohol use had been reported in all age groups including among children aged 10-17 years. The same report further states that, in the state of Uttarakhand, 38.1% of males and 18.8% of the total population used alcohol, and about 1.4% population used cannabis (charas or ganja); remarkable gender differences existed in the pattern of alcohol use; while 27.3% of men used alcohol, the corresponding figure for women was just 1.6%. Further, about one in five alcohol-using men suffer from alcohol dependence, while only one in sixteen alcohol-using women are dependent on it.

The substance abuse patterns and their epidemiological correlates are affected significantly, if not largely, by regional variations.

Recommendations

There is an urgent need to ensure quality enhancement of the services and facilities in the state as the programs/modules for de-addiction/rehabilitation used in practice, mostly by recovering addicts/alcoholics, are not standardized and mostly arbitrary. Private de-addiction centers need effective regulatory & supportive guidance from the concerned Department of State administration. It is also felt that the provision of in-facility treatment of chemical dependency must consider the financial limitations of the substance abusers. Feedback should be taken from the users at the private de-addiction centers and their relatives about their experience during their stay, and programs/models for de-addiction and rehabilitation should involve the afflicted families too; they should provide them with support in the line of self-help family systems groups like alcoholics anonymous.

Limitations

The selection of study samples was from in-facility clients or those coming for treatment only, so the study results may possibly differ from that of a community-based study. The findings of our study may thus be possibly different from the true picture of drug use in the community and cannot be broadly generalized. The study was also limited in the sense that clients were exclusively males, and therefore the results may not be extrapolated to female clients. However, it could truly depict the gender-based variation in admission rates of patients to the de-addiction centers.

## Conclusions

Very few state‑level studies have been strategically done to reflect the prevalence of various types of substance use in Uttarakhand. Results of the current study will help in making drug policies and management guidelines, including prioritizing the importance of the establishment of de‑addiction cum rehabilitation centers at district levels to deal with the problem more effectively and also encourage further studies.
